# Following the science? Comparison of methodological and reporting quality of covid-19 and other research from the first wave of the pandemic

**DOI:** 10.1186/s12916-021-01920-x

**Published:** 2021-02-23

**Authors:** Terence J. Quinn, Jennifer K. Burton, Ben Carter, Nicola Cooper, Kerry Dwan, Ryan Field, Suzanne C. Freeman, Claudia Geue, Ping-Hsuan Hsieh, Kris McGill, Clareece R. Nevill, Dikshyanta Rana, Alex Sutton, Martin Taylor Rowan, Yiqiao Xin

**Affiliations:** 1 Institute of Cardiovascular and Medical Sciences, University of Glasgow, New Lister Building Campus, Alexandra Parade, Glasgow, G31 2ER UK; 2 Institute of Cardiovascular and Medical Sciences, University of Glasgow, Glasgow, UK; 3 Institute of Psychiatry, Psychology and Neuroscience Kings College London, London, UK; 4 Department of Health Sciences, University of Leicester, Leicester, UK; 5 Cochrane Methods Support Unit, Cochrane, UK, Oxford, UK; 6 Health Economics and Health Technology Assessment, University of Glasgow, Glasgow, UK; 7 Tri-Service General Hospital, National Defence Medical Center, Taipei, Taiwan; 8 NMAHP Research Unit, Glasgow Caledonian University, Glasgow, UK

**Keywords:** Clinical trials, COVID-19, Methodology, Observational research, Publishing, Reporting

## Abstract

**Background:**

Following the initial identification of the 2019 coronavirus disease (covid-19), the subsequent months saw substantial increases in published biomedical research. Concerns have been raised in both scientific and lay press around the quality of some of this research. We assessed clinical research from major clinical journals, comparing methodological and reporting quality of covid-19 papers published in the first wave (here defined as December 2019 to May 2020 inclusive) of the viral pandemic with non-covid papers published at the same time.

**Methods:**

We reviewed research publications (print and online) from *The BMJ*, *Journal of the American Medical Association* (*JAMA*), *The Lancet*, and *New England Journal of Medicine*, from first publication of a covid-19 research paper (February 2020) to May 2020 inclusive. Paired reviewers were randomly allocated to extract data on methodological quality (risk of bias) and reporting quality (adherence to reporting guidance) from each paper using validated assessment tools. A random 10% of papers were assessed by a third, independent rater. Overall methodological quality for each paper was rated high, low or unclear. Reporting quality was described as percentage of total items reported.

**Results:**

From 168 research papers, 165 were eligible, including 54 (33%) papers with a covid-19 focus. For methodological quality, 18 (33%) covid-19 papers and 83 (73%) non-covid papers were rated as low risk of bias, OR 6.32 (95%CI 2.85 to 14.00). The difference in quality was maintained after adjusting for publication date, results, funding, study design, journal and raters (OR 6.09 (95%CI 2.09 to 17.72)). For reporting quality, adherence to reporting guidelines was poorer for covid-19 papers, mean percentage of total items reported 72% (95%CI:66 to 77) for covid-19 papers and 84% (95%CI:81 to 87) for non-covid.

**Conclusions:**

Across various measures, we have demonstrated that covid-19 research from the first wave of the pandemic was potentially of lower quality than contemporaneous non-covid research. While some differences may be an inevitable consequence of conducting research during a viral pandemic, poor reporting should not be accepted.

**Supplementary Information:**

The online version contains supplementary material available at 10.1186/s12916-021-01920-x.

## Background

The severe acute respiratory syndrome coronavirus 2 (SARS COV-2), and the resulting clinical coronavirus disease 2019 (covid-19) have disrupted all aspects of healthcare [1]. As the pathogen is new, in the first wave of viral infections, research was urgently needed to influence policy and practice. Although we have made substantial progress, there is still much we do not know about the virus and the necessary research spans many methodological approaches including observational epidemiology, assessment of the accuracy of test strategies, trials of interventions and many others.

The main platform for sharing results of scientific research remains the peer reviewed, biomedical journal. Biomedical publishers reported a substantial increase in submissions in early 2020, with most of the content related to covid-19. Journals responded to the increasing volume of covid-19 research with rapid publication of these papers [2, 3]. However, there is concern that in the rush to share data, some good practice aspects of research design, conduct and interpretation may have been lost [4]. Arguments around the integrity and quality of covid-19 research have been rehearsed in the lay and scientific press [5, 6]. While these important issues have generated substantial copy, there has been little quantitative, scientific description of research quality as a basis for this discourse.

There are many aspects of a scientific paper that contribute towards the overall ‘quality’ [7]. Two of the most important are the design and conduct of the research (methodological quality) and the way the study and results are communicated (reporting quality) [8, 9]. These aspects of scientific process are complementary. Methodological quality ensures that the results presented are robust and biases are minimised, while reporting quality ensures transparency and aids interpretation.

We aimed to assess methodological quality (risk of bias) and reporting quality (compliance with reporting guidelines) for papers published in the highest impact biomedical journals during the first wave (here defined as December 2019 to May 2020 inclusive) of the covid-19 pandemic. We compared results for covid-19 and contemporaneous non-covid research.

## Methods

Although not a systematic review in the classical sense, where appropriate we adhered to the relevant sections of the Preferred Reporting Items for Systematic reviews and Meta-analyses (PRISMA) guidance [10]. No ethical approvals were required. Full details on methods are available in the study protocol and additional files (Additional Files 1: Supplementary text S1–3).

There was no funding source for this study. The corresponding author had full access to all the data in the study and had final responsibility for the decision to submit for publication.

### Search strategy

We included the following journals, chosen as representing the highest impact clinical research titles in the category of ‘Medicine, General and Internal’ (based on Journal Citation Reports 2018 category (Clarivate Analytics)): *The BMJ* (British Medical Association), *The Journal of the American Medical Association* (*JAMA*, American Medical Association), *The Lancet* (Elsevier) and *The New England Journal of Medicine* (*NEJM*, Massachusetts Medical Society). We favoured these four titles as they publish weekly print editions, have international readerships and are generally considered to have the highest publication standards. These were also some of the first journals to publish clinical research on covid-19 during the first wave of the pandemic.

Each print journal was hand searched by a single reviewer, beginning from January 2020 to identify the first clinical covid-19 research papers. These publications were the inception point for further hand searching to collate all research papers from that journal. As an internal validity check, the journals’ online search facility and/or any specific covid-19 resource hubs were checked to ensure no relevant content had been missed. Paper selection was from the first week that the relevant journal published a covid-19 related clinical research paper and concluded on Sunday 17th of May inclusive. On this date, journal websites were searched for all covid-19 papers available online prior to print. These dates were chosen to represent the first wave of the covid-19 pandemic.

### Inclusion and exclusion criteria

We assessed all articles labelled as original clinical research, including Brief Reports. We did not include non-research content such as editorials or commentary. Within the original clinical research remit, we excluded papers that were not suitable for assessment with our chosen quality tools, for example pre-clinical or translational science. Decisions on exclusion were made by raters as part of the initial assessment of potentially eligible papers.

We classified included papers based on the primary study method and this classification informed the choice of the quality assessment tools used. We pre-defined six categories chosen to encompass the most commonly used clinical research designs: diagnostic test accuracy; observational studies (subdivided into case-study/case-series, case-control, cohort and cross-sectional); prognosis; qualitative; randomised controlled trials (RCT); and systematic review.

We created two groups to facilitate comparisons, ‘covid-19’ research and ‘non-covid’. The covid-19 label was applied where the exposure, intervention, test or outcomes related to covid-19/SARS COV-2.

### Risk of bias and reporting

We assessed the two quality measures (risk of bias and reporting) separately, using validated tools suited to our pre-specified study designs [10–21]. Choice of tool was based on published validation, availability of training materials and guidance. We favoured those tools used by Cochrane [22] and featured on the EQUATOR resource (Enhancing the QUAlity and Transparency Of health Research) [23] where possible. For RCTs, we used the Cochrane risk of bias [11] tool and the Consolidated Standards of Reporting Trials (CONSORT) checklist [12]. For observational studies, we used the National Heart Lung and Blood Institute tool (NHLBI) [13] and the Strengthening the Reporting of Observational Studies in Epidemiology (STROBE) [14](Table 1). To facilitate comparisons where general and specialist guidance was available, we used the more general checklists (Table 1).

**Table 1:** Tools used to assess quality (risk of bias) and reporting

Design	Quality (risk of bias)	Domains assessed	Reporting
Controlled trial	Cochrane RoB	Randomisation, allocation, blinding (participants), blinding (outcomes), incomplete outcomes, selective reporting, other	CONSORT
Observational	NHLBI	Question, population, exposure, outcomes, confounding, other	STROBE
Test accuracy	QUADAS2	Patient selection, index test, reference standard, flow and timing, generalisability, other	STARD
Systematic review	AMSTAR2	Design and protocol, search strategy, paired extraction, inclusion/exclusion, risk of bias, meta-analysis, conflicts of interest, other	PRISMA
Qualitative	CASP	Design, recruitment, data collection, relationships, analysis, other	COREQ
Prognosis	PROBAST	Participants, predictors, outcomes, analysis, generalisability, other	TRIPOD

For methodological quality assessment, we assessed risk of bias at the level of pre-specified domains and at the level of the complete paper with a final overall grading of ‘low’, ‘high’ or ‘unclear’ risk of bias. Decisions on overall risk of bias were made by the rater pairs, informed by the domain level assessments. Overall risk was not defined by a threshold of number of individual domains that scored low or high risk; rather, overall risk of bias was considered on a paper by paper basis.

For reporting quality, we assessed adherence with reporting guidance at an individual item level with reporting scored as ‘yes’ (where reporting was deemed adequate), ‘no’ or ‘not applicable’. This allowed calculation of proportional adherence (percentage of total items reported) as the number of adequately reported items against the number of relevant items for the paper.

### Data extraction

The team were all researchers with experience and training in meta-research. From a pool of 12 reviewers, we created six reviewer pairs, consisting of one experienced and one less experienced researcher. Each pair had recourse to the senior author (TQ) for advice or where there was disagreement on assessment. Assessor pairs were allocated a test set of six papers (covid-19 and non-covid) for review and calibration within pairs and within the group. Pairs were then randomly allocated eligible papers in blocks. Reviewer pairs extracted descriptive and outcomes data independently and compared results. In the case of disagreement, papers were discussed with a third reviewer (the experienced reviewer from another reviewer pair). As a validity check, a random selection of 10% of the included papers was selected for further review by an independent reviewer (an experienced reviewer from the pool of reviewers, that was not part of the respective reviewer pair). We collected data on domain level disagreement requiring discussion for each assessor pair to calculate percentage disagreement within the group. All random allocation used the random.org online resource [24].

Reviewers used standardised data extraction forms, piloted on two studies (one RCT, one observational) [25, 26]. We collated the following study-level details: journal, study design, whether the paper was identified as a ‘Brief Report’ or equivalent, the exposure of interest (or intervention, or index test), covid-19 status, the total ‘n’ included in the study at baseline (for a systematic review this was taken as the number of included papers), follow-up (time from first measure to last measure for primary outcome, quantified in weeks) and funding source (academic or industry). We assessed the timing of publication, comparing papers published before a midpoint of April 12, 2020 (10 weeks after the date of the first covid-19 publication), to publication after this date. We assessed whether the paper was framed as having a positive or neutral result (using a method described previously [27]), had an accompanying editorial, or had retraction or serious correction (full definitions in Additional File 1: Supplementary text S3).

### Outcomes

Our primary outcome was methodological quality, based on assessment of overall risk of bias for each included paper. Our co-primary outcome was reporting quality, based on adherence to reporting guidelines and quantified as proportion of relevant reporting items completed for each included paper.

### Data synthesis and analyses

We locked the database on 11th of June when the last review was submitted, and quality control checks were complete. A statistician independent of the main review group conducted the analyses. Covid-19 status was numerically coded so that this variable was not obvious to the statistician.

We tabulated descriptive statistics for covid-19 and non-covid research, comparing features of the included papers using non-parametric and proportional statistics as required. We collated data on inter-rater agreement from reviewer pairs to calculate summary reliability for the team.

We created graphical illustrations of methodological and reporting quality at paper level and in aggregate [28]. We created modified star plots, to describe domain level and overall risk of bias and reporting adherence for covid-19 and non-covid papers. For each domain, we calculated difference in score between covid-19 and non-covid with corresponding uncertainty (95% confidence interval [95%CI]) using an approach that accounted for small samples sizes [29].

We compared proportions of ‘low risk of bias’ in covid-19 and non-covid research across all included papers. We fitted a mixed effects logistic regression describing the odds of study level low risk of bias, where the rater pairs were fitted as a random effect to control for the heterogeneity introduced by scorers. The multivariable analysis was adjusted for time of publication, funder (academic or industry), study results (positive or negative), study design and journal. Stata version 15 (StataCorp) was used for the primary quantitative analyses.

## Results

**Fig. 1:**
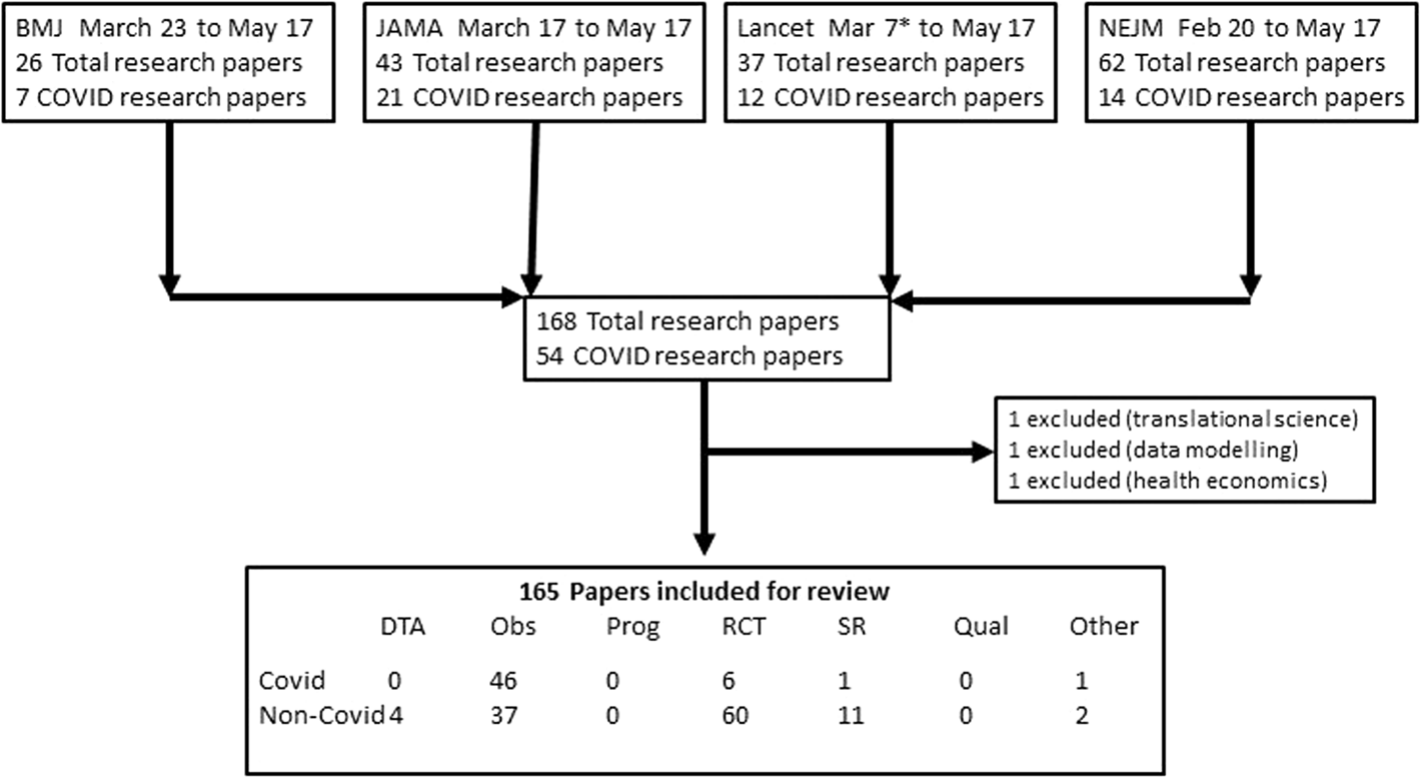
PRISMA flow diagram describing search strategy and inclusion. Flow diagram illustrating literature search and results. NB The Lancet published two data modelling covid-19 research papers in February 2020; these did not meet our definition of clinical research and so first paper included was March

**Fig. 2:**
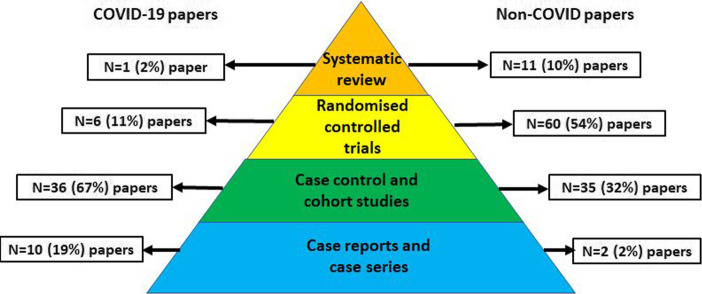
Comparing published covid-19 and non-covid research using the classical ‘evidence pyramid’ hierarchy. NB For this illustration, the category ‘observational’ is further divided into cases series and case control/cohort. Not all of our chosen research designs feature on the classical evidence hierarchy pyramid. All differences are significant at *P* < 0.05

We assessed 168 titles and included 165 research papers (Fig. 1). The research method differed between covid-19 and non-covid papers. Covid-19 papers were less likely to be based on RCTs (*n* = 6 (11%) for covid-19 v *n* = 60 (53%), difference 42% (95%CI 28 to 53)) and more likely to use case series or other observational designs (*n* = 46 (85%) for covid-19 papers v *n* = 37 (32%), difference 52% (95%CI 37 to 63)) (Fig. 2).

Covid-19 papers were more likely to be published in Brief Report format (30% for covid-19 v 5%, difference 25% (95%CI 13 to 38)), to have an accompanying editorial (57% for covid-19 v 38%, difference 18% (95%CI 2 to 33)) and to have a retraction or major post-publication correction (13% for covid-19 v 0%, difference 13% (95%CI 6 to 24)). Covid-19 papers had smaller sample size (median 96 participants for covid-19 v 815 participants, *P* < 0.0001) and shorter follow-up (median 4 weeks for covid-19 v 52 weeks, P < 0.0001) (Table 2).

**Table 2:** Comparing paper level characteristics of published covid-19 and non-covid research

	COVID-19 papers ***n*** = 54	Non COVID papers ***n*** = 114
**Method,** ***n*** **(%)**		
Randomised controlled trial	6 (11%)	60 (53%)
Observational	46 (85%)	37 (32%)
Systematic reviews	1 (2%)	11 (10%)
Test accuracy	0 (0%)	4 (4%)
Other	1 (2%)	2 (2%)
**Content median (IQR)**		
Total number included	96 (16.5 to 762)	815 (219 to 4893)*
Follow-up (weeks)	4 (3 to 7)	52 (28 to 116)*
Positive result, *n* (%)	7 (13%)	74 (65%)*
Industry funding, *n* (%)	2 (4%)	31 (27%)*
**Post-publication,** ***n*** **(%)**		
Brief report format	16 (30%)	6 (5%)*
Editorial	31 (57%)	43 (38%)**
Correction/retraction	7 (13%)	0 (0%)*

*Difference *p* < 0.0001

**Difference *p* < 0.05

Agreement within rater pairs was 85% for studies assessed using the Cochrane risk of bias tool, 92% CONSORT, 86% NHLBI observational research tool, 90% STROBE.

Aggregate and domain level, risk of bias charts and domain level modified star charts for RCTs and observational studies are presented in Figs. 3a,b and 4a,b and Additional File 1: Figs. S1-S10.

**Fig. 3:**
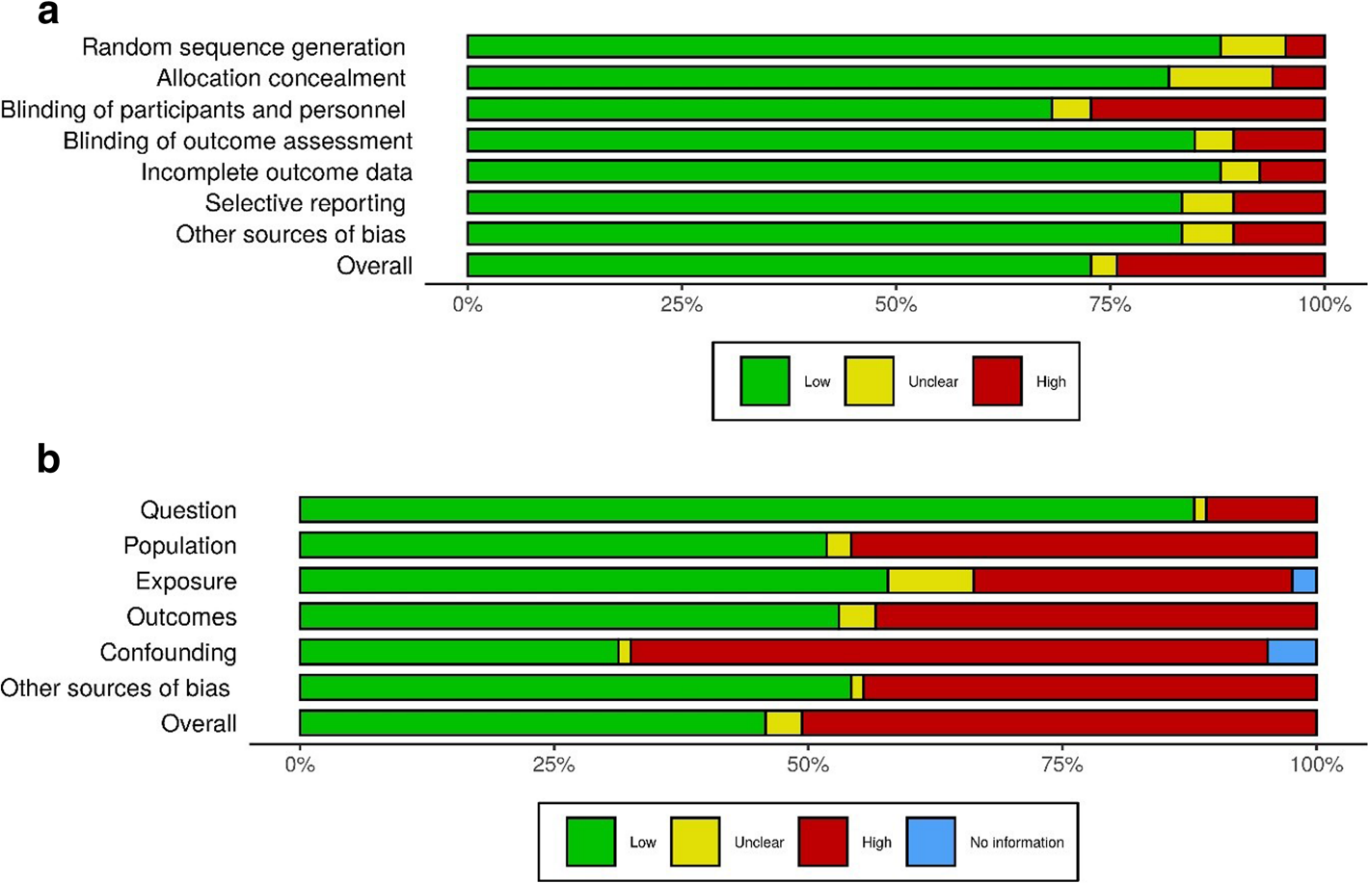
**a**, **b** Risk of bias charts

**Fig. 4:**
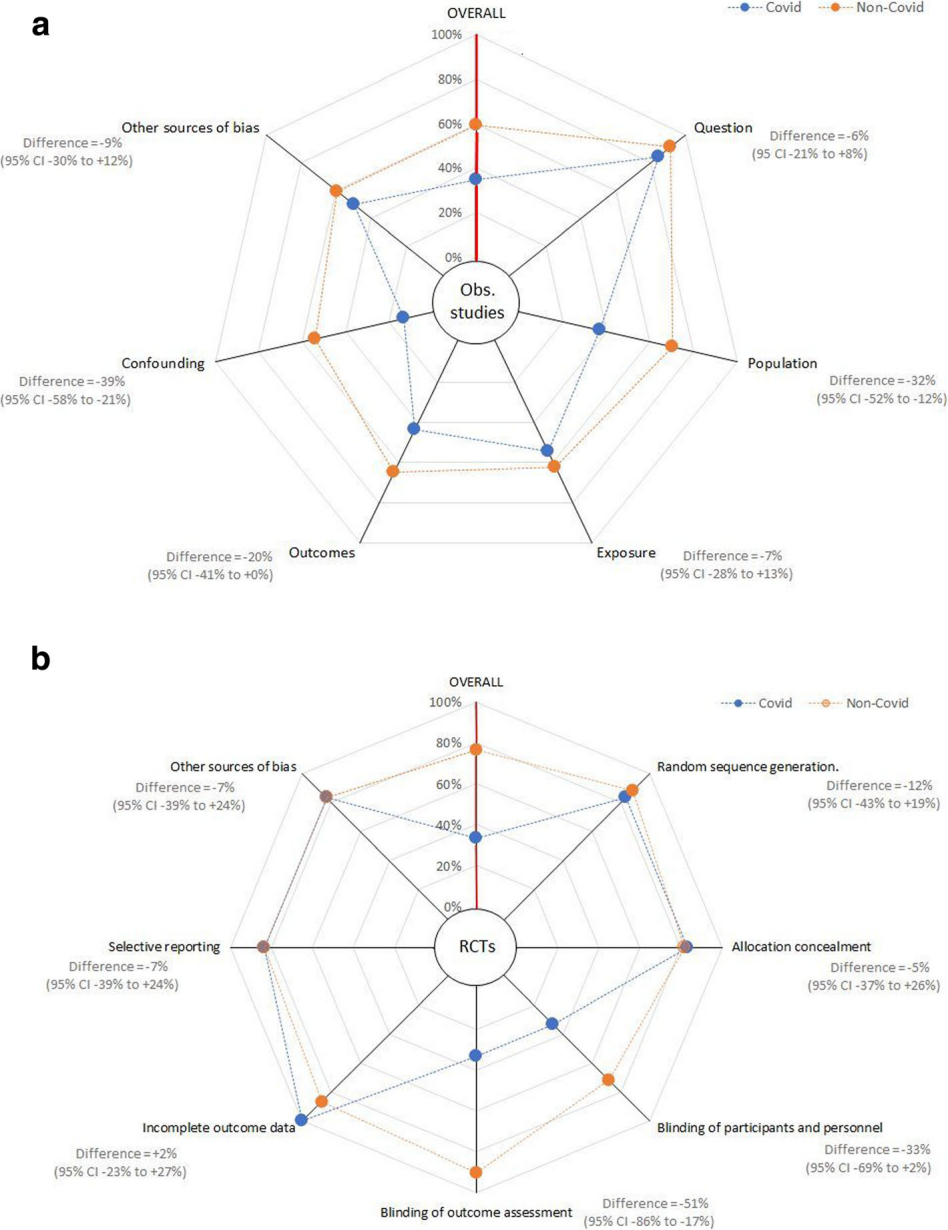
**a**, **b** Modified star plots, describing methodological quality (risk of bias) overall and for each domain of the risk of bias assessment tool for differing study methods. **a** Randomised controlled trials (using Cochrane RoB1 tool). **b** Observational studies (using NHLBI tool). Blue spokes represent covid-19 studies and orange spokes represent non-covid 19 studies. *RCTS*, randomised controlled trials; *Obs studies*, observational studies

### Outcome 1: Methodological quality

Overall 101 of 165 studies (61%, 95%CI 50–74) were rated as a low risk of bias. Of the non-covid studies, 83 (73%, 95%CI 64 to 81) were rated as low risk of bias, compared to 18 (34%, 95%CI 22 to 48) in the covid-19 group (difference 65%, 95%CI 50–75).

Covid-19 study status was associated with study-level risk of bias. Low risk of bias was 6 times more common in the non-covid group than the covid-19 group (OR 6.3, 95%CI 2.9 to 14.0; *p* < 0.001). Bias differed between study designs. Compared to observational designs, RCTS were almost three times more likely to have low risk of bias (OR 3.0, 95%CI 1.4 to 6.2; *p* = 0.004). The time of publication, the journal of publication, the study design, study findings and source of funding were all not associated with study level risk of bias.

In multivariable analysis, covid-19 papers remained associated with almost six times higher risk of bias (aOR = 6.1, 95%CI 2.1–17.7; *p* = 0.001) with none of the other covariates exhibiting evidence of association with risk of bias.

Differences were still evident when analyses were restricted to RCTs or observational studies only. For RCTs, low risk of bias was more common in non-covid papers (46 papers, 77%) than covid-19 papers (1 paper, 17%) (difference 60%, 95%CI 19–76). Low risk of bias in observational studies was also more common in non-covid studies (23 papers, 61%) than in covid-19 studies (15 papers, 32%) (difference 29%, 95%CI 8–47).

All domains for observational studies and all but one domain for RCTs suggested lower quality scores in the covid-19 papers, although with small sample sizes not all reached significance at the conventional level (Figs. 3a,b and 4a,b).

### Outcome 2: Reporting quality

Proportional adherence to reporting guidelines differed between covid-19 and non-covid research. Mean percentage adherence to reporting guidance for non-covid was higher (84%, 95%CI 81 to 87) than for covid-19 studies (71%, 95%CI 66 to 77), with a mean difference of 13% (95%CI 0 to 27).

In analyses restricted to RCTs (CONSORT) and observational studies (STROBE), differences between covid-19 and non-covid research were apparent but did not reach statistical significance. For RCTs, mean adherence to reporting guidelines for non-covid studies was 90% and for covid-19 papers this was 87% (difference 3%, 95%CI − 12 to 43). For observational studies, mean adherence for non-covid studies was 75% (95%CI 69 to 81) and for covid-19 studies was 69% (95%CI 63 to 75) (difference 6%, 95%CI − 14 to 24).

All reporting domains for observational studies suggested lower quality scores in the covid-19 papers, differences were less obvious for RCT reporting and small sample sizes preclude meaningful comparative testing at this level (Additional File 1: Figs. S9–10).

## Discussion

In our analysis of 168 clinical research papers published across the four highest impact medical journals during the first wave of the pandemic, we found that papers with a covid-19 focus differed from other published research in many ways—smaller sample size, shorter follow-up and greater proportion of case-series and other observational designs. Some of these differences are understandable for research describing the first wave of a novel pathogen and the research community has learned much about the virus since we completed our review. More concerning was the suggestion of poorer methodological quality (risk of bias) and poorer reporting of the published covid-19 research. Given the time pressures to produce data and the novelty of the virus, some pragmatism can perhaps be allowed around research design, for example it seems intuitive that the first papers describing a new pathogen are more likely to be descriptive. However, there is no reason to sacrifice comprehensive, transparent reporting.

In a time-sensitive publishing space, some may argue that publication standards may have been relaxed for publications on covid-19 during the first wave, with the anticipation that later papers would be more robust. We would argue that there is a scientific and ethical imperative to maintain standards of conduct and reporting. We explored the effect of time, and within our first wave sampling frame, we found that publication date did not have a major effect on quality. Many of the covid-19 papers were published as ‘Brief Report’ format. Limits on word count could compromise quality, but we did not find that format of submission explained variation in scores. Planning, conducting and reporting a trial take time and it seems intuitive that early covid-19 research may favour observational designs. However, our finding of lower quality in covid-19 was not solely driven by an excess of early observational studies. Observational studies were seen throughout the period of assessment, and within those observational papers, quality was poorer for covid-19 papers. Regardless our measures of quality and reporting were bespoke to the trial design and the imbalance of trials to observational data for covid-19 research will not fully explain the findings.

To ensure a platform for the important emerging research, standards for covid-19 submissions may have been relaxed compared to non covid-19 submissions. It could be argued that we should be more tolerant of potential risk of bias when assessing covid-19 studies. We acknowledge that covid-19 research was at an early stage when we performed our review and methods may have been modified accordingly. For example, given the uncertain nature of covid-19 and potential treatments, full blinding may have been considered too risky by investigators and for observational studies it would be difficult to know the most important confounder variables to correct for in covid-19 analyses.

### Strengths and limitations

We followed best practice guidance for meta-research and used validated and objective scoring tools. Within the time constraints of this rapid study, we created training and calibration processes to ensure consistency in assessment. It would not have been possible to mask our raters to the covid-19 status of the papers under review. Consciously or subconsciously, raters may have preferentially marked down covid-19 research. To mitigate this risk, we embedded multiple checks of internal validity throughout the review process.

Our choice of quality assessment tool was based on explicit criteria and agreed by the group but we recognise that there are a variety of tools available with no consensus on the preferred approach [8]. While our chosen tools were appropriate for most published studies, they were not always completely aligned with the included study designs. For example, in our systematic review category we used the generic AMSTAR (Assessing the Methodological Quality of Systematic reviews) [17] and PRISMA (Preferred Reporting Items for Systematic reviews and Meta-analyses) [10] tools to assess reviews using network meta-analyses, individual patient data and living systematic review designs, all of which have their own quality assessment guidance [30–32].

A selection bias is possible. We did not attempt a comprehensive analysis of all clinical research published in the first wave of the pandemic, as this would involve thousands of papers. Rather we selected exemplar, high profile, weekly clinical journals who had published substantial numbers of covid-19 research papers. Although the early covid-19 research was often shared with these journals, we recognise that our included papers represent only a fraction of the totality of covid-19 research published in the first wave.

Our study followed a pre-specified and publicly available protocol and was designed to assess a selection of covid-19 papers published during the first wave of the pandemic. Other approaches to an assessment of methodological and reporting quality are plausible. For example, we anticipated an imbalance of trials to observational research methods in covid-19 and non-covid research and did not attempt a matched case-control design where the only difference was the covid-19 subject matter. It could be argued that the ideal method to assess the quality of covid-19 research would be to compare the covid-19 papers with contemporaneous research describing an established but similarly infectious and dangerous viral agent. While theoretically possible, these designs would have been almost impossible to deliver in practice during the first wave.

Quality of science is more than just valid methods and transparent reporting. Indeed, in the covid-19 context, other important factors like inclusiveness, data sharing and clinical urgency may take precedence over rigid rules on method and reporting. Even within our chosen remit of methodological quality, there are many facets. Some of our tools have a strict focus on internal validity (bias) while others also consider external validity (generalisability). Accepting all this, we do not claim to have described a definitive measure of overall study quality, but we have described and quantified fundamental aspects of conduct and reporting that should be maintained in all scientific publications. We believe this is especially true when the topic matter is so important to health and society.

### Research in context

The volume of published clinical research is constantly increasing, even more so, if we consider the pre-print servers. It would not have been possible to assess the entirety of the biomedical literature published during the first wave of the pandemic. We limited to those journals with the greatest clinical impact and considered to have the highest standards. If there are concerns around quality in these four flagship journals, it seems likely these issues will also permeate other journals. There is research to support this view, studies of research methods, for example prognostic modelling [33] and pharmacoepidemiology [34], or research populations, for example older adults [35], have all reported methodological concerns in the covid-19 literature.

The story of hydroxychloroquine as a potential covid-19 treatment is a pertinent example of the need for rapid communication of science and the attendant risk of sharing potentially biased research. Based on predominantly observational studies, the drug achieved substantial visibility and entered clinical practice [36]. Further observational studies suggested the benefits may have been overstated and large RCTs now suggest the drug is harmful [37]. Methodology and reporting were both major factors in the rise and fall of this drug [38]. Recent retractions and corrections of covid-19 hydroxychloroquine papers [39] also highlight a limitation in the scope of our approach. Our assessment of quality can only include what is published and available to the public. Even the best quality assessment tools will not pick up those cases where there have been deliberate or inadvertent errors in the research process.

Quality concerns are not an exclusive covid-19 phenomenon, and it is worth noting that our non-covid papers also had biases. Issues with reporting, which were prevalent in both covid-19 and non-covid studies, seem less forgivable. Although differences in reporting quality were not of the same magnitude as differences in methodological quality, if anything, where research is produced at speed to answer an urgent clinical question, then the need for comprehensive and fully transparent reporting is even greater. All the journals assessed in this analysis adhere to CONSORT recommendations for RCTs and completion of a CONSORT checklist is mandatory. Experience tells us that mandating a checklist is not a panacea. Since the introduction of CONSORT, there have been improvements in trial reporting, but poor reporting remains prevalent in contemporary research [40, 41].

### Future research

We offer only a snapshot of the covid-19 evidence base from the first wave of the pandemic. Our analyses of the effects of time are limited by the relatively short period within which we selected papers. As we move into second wave of the pandemic and beyond, it would be interesting to repeat our analysis looking for longer term temporal trends in quality. Even within the period of this review, new concerns around research publications are emerging. Using our approach to look at pre-prints, inclusiveness or data sharing arrangements would all be informative. We assessed various factors that may be associated with research quality, but recognise that many other plausible factors exist, some specific to covid-19 and some common to all clinical research [42].

## Conclusion

Covid-19 research published in major journals during the first wave of the pandemic had methodological and reporting issues that ultimately compromise the utility of the research and may cause harm. The clinical response to covid-19 has seen many examples of sacrifice and tragedy. In clinical science we must not sacrifice research quality in the race to publish data and it would be further tragedy if current researchers and publishers do not learn from the first wave of covid-19 research.

## Supplementary Information

**Additional file 1: Supplementary text S1**. Description of Study team. **Supplementary text S2**. Categories of research design assessed in the project. **Supplementary text S3**. Data dictionary for ‘included studies’ master sheet. **Figure S1-S4** Traffic light visual summary of risk of bias (ROB). **S1a**) Traffic light summary of ROB for RCTs at individual study level. **S1b**) Traffic light summary of ROB for RCTs as aggregate scores. **S2a**) Traffic light summary of ROB for observational studies at individual study level. **S2b**) Traffic light summary of ROB for observational studies at aggregate level. **S3a**) Traffic light summary of ROB for test accuracy studies at individual study level. **S3b**) Traffic light summary of ROB for test accuracy studies at aggregate level. **S4a**) Traffic light summary of ROB for systematic reviews at individual study level. **S4a**) Traffic light summary of ROB for systematic reviews at aggregate level. **Figure S5**. Reporting guideline adherence (CONSORT) for RCTs. **Figure S6**. Reporting guideline adherence (STROBE) for observational studies. **Figure S7** Reporting guideline adherence (STARD) for test accuracy studies. **Figure S8** Reporting guideline adherence (PRISMA) for systematic review. **Figure S9** Modified star plot describing overall and individual item level reporting adherence for STROBE (observational) reporting. **Figure S10** Modified star plot describing overall and individual item level reporting adherence for CONSORT (RCT) reporting.

## Data Availability

Following publication data will be freely available on the NIHR CRSU website.

## Abbreviations

AMSTAR: Assessing the Methodological Quality of Systematic reviews
CONSORT: Consolidated Standards of Reporting Trials
Covid-19: Coronavirus disease 2019
EQUATOR: Enhancing the QUAlity and Transparency Of health Research
JAMA: The Journal of the American Medical Association
NHLBI: National Heart Lung and Blood Institute tool
NEJM: The New England Journal of Medicine
OR: Odds ratio
PRISMA: Preferred Reporting Items for Systematic reviews and Meta-Analyses
RCT: Randomised controlled trial
SARS COV-2: Severe acute respiratory syndrome coronavirus 2
STROBE: Strengthening the Reporting of Observational Studies in Epidemiology
