# Correction to: Following the science? Comparison of methodological and reporting quality of covid-19 and other research from the first wave of the pandemic

**DOI:** 10.1186/s12916-021-02073-7

**Published:** 2021-08-22

**Authors:** Terence J. Quin, Jennifer K. Burton, Ben Carter, Nicola Cooper, Kerry Dwan, Ryan Field, Suzane C. Freeman, Claudia Gue, Ping-Hsuan Hsieh, Kris McGill, Clareece R. Nevill, Dikshyanta Rana, Alex Sutton, Martin Taylor Rowan, Yiqiao Xin

**Affiliations:** 1 Institute of Cardiovascular and Medical Sciences, University of Glaslow, New Lister Building Campus, Alexandra Parade, Glaslow, G31 2ER UK; 2 Institute Cardiovascular and Medical Sciences, University of Glaslow, Glaslow, UK; 3 Institute of Psychiatry, Psychology and Neuroscience Kings College London, London, UK; 4 Department of Health Sciences, University Leicester, Liecester, UK; 5 Cochrane Methods Support Unit, Cochrane UK, Oxford, UK; 6 Health Ecocomics and Health Technology Assessment, University of Glaslow, Glaslow, UK; 7 Tri-Service General Hospital, National Defence Medical Center, Taipei, Taiwan; 8 NMAHP Research Unit, Glaslow Caledonian University, Glaslow, UK

**Correction to: BMC Medicine 19, 46 (2021)**

**https://doi.org/10.1186/s12916-021-01920-x**

The original article [1] contained an affiliation error for co-author, Dikshyanta Rana which has since been corrected.
