# Supplementary material for: Following the science? Comparison of methodological and reporting quality of covid-19 and other research from the first wave of the pandemic
Source: BMC Med. 2021 Feb 23;19:46. doi: 10.1186/s12916-021-01920-x (PMC7899793; doi:10.1186/s12916-021-01920-x)
Supplement: Supplementary file 1 — Additional file 1: Supplementary text S1. Description of Study team. Supplementary text S2. Categories of research design assessed in the project. Supplementary text S3. Data dictionary for ‘included studies’ master sheet. Figure S1-S4 Traffic light visual summary of risk of bias (ROB). S1a) Traffic light summary of ROB for RCTs at individual study level. S1b) Traffic light summary of ROB for RCTs as aggregate scores. S2a) Traffic light summary of ROB for observational studies at individual study level. S2b) Traffic light summary of ROB for observational studies at aggregate level. S3a) Traffic light summary of ROB for test accuracy studies at individual study level. S3b) Traffic light summary of ROB for test accuracy studies at aggregate level. S4a) Traffic light summary of ROB for systematic reviews at individual study level. S4a) Traffic light summary of ROB for systematic reviews at aggregate level. Figure S5. Reporting guideline adherence (CONSORT) for RCTs. Figure S6. Reporting guideline adherence (STROBE) for observational studies. Figure S7 Reporting guideline adherence (STARD) for test accuracy studies. Figure S8 Reporting guideline adherence (PRISMA) for systematic review. Figure S9 Modified star plot describing overall and individual item level reporting adherence for STROBE (observational) reporting. Figure S10 Modified star plot describing overall and individual item level reporting adherence for CONSORT (RCT) reporting. [file 12916_2021_1920_MOESM1_ESM.docx]

**Supplementary Materials for paper: Methodological and reporting quality of COVID-19 and other research**

Study protocol is available online: <http://www.nihrcrsu.org/publications%20and%20workshops/protocols_publications_and_presentations/>

and submitted as additional Supplemental Materials

PRISMA checklist submitted as additional Supplemental Materials

**Contents**

**Study team 3**

**Categories of research design assessed in the project 4**

**Data dictionary for ‘included studies’ master sheet 5**

**Description of visual summaries of risk of bias 6**

**Risk of bias for randomised controlled trials 1a) Individual study level 7**

**Risk of bias for randomised controlled trials 1b) Aggregate 8**

**Risk of bias for observational studies 2a) Individual study level 9**

**Risk of bias for observational studies 2b) Aggregate 10**

**Risk of bias for test accuracy studies 3a) Individual study level 11**

**Risk of bias for test accuracy studies 3b) Aggregate 11**

**Risk of bias for systematic reviews 4a) Individual study level 12**

**Risk of bias for systematic reviews 4b) Aggregate 13**

**Reporting guideline adherence (CONSORT) for randomised controlled trials 13**

**Reporting guideline adherence (STROBE) for observational studies 14**

**Reporting guideline adherence (STARD) for test accuracy studies 15**

**Reporting guideline adherence (PRISMA) for systematic review 16**

**Modified star plot for STROBE (observational) reporting 17**

**Modified star plot for CONSORT (RCT) reporting 18**

**Additional File 1. Supplementary text S1.**

**Study team**

Suzanne C Freeman – Department of Health Sciences, University of Leicester

0000-0001-8045-4405

Clareece R Nevill – Department of Health Sciences, University of Leicester

0000-0001-8305-2516

Alex Sutton - Department of Health Sciences, University of Leicester

0000-0002-8934-9940

Nicola Cooper - Department of Health Sciences, University of Leicester

0000-0002-4486-2791

Kris McGill – NMAHP Research Unit, Glasgow Caledonian University

0000-0002-0307-1440

Kerry Dwan – Cochrane Methods Support Unit

0000-0001-6918-1215

Jennifer K Burton - Institute of Cardiovascular and Medical Sciences, University of Glasgow

0000-0002-4752-6988

Martin Taylor Rowan – Institute of Cardiovascular and Medical Sciences, University of Glasgow

Terence J Quinn - Institute of Cardiovascular and Medical Sciences, University of Glasgow

0000-0003-1401-0181

Ryan Field – Health Economics and Health Technology Assessment, University of Glasgow

0000-0002-4424-9890

Ping-Hsuan Hsieh - Health Economics and Health Technology Assessment, University of Glasgow; Tri-Service General Hospital, National Defense Medical Center, Taipei, Taiwan.

0000- 0002- 0430- 260X

Claudia Geue – Health Economics and Health Technology Assessment, University of Glasgow

0000-0003-2243-0733

Dikshyanta Rana - Health Economics and Health Technology Assessment, University of Glasgow

0000-0001-9133-3094.

Yiqiao Xin – Health Economics and Health Technology Assessment, University of Glasgow

0000-0001-5856-3103

**Statistician:** Ben Carter – Institute of Psychiatry, Psychology and Neuroscience Kings College London

0000-0003-0318-8865

**Additional File 1. Supplementary text S2.**

**Categories of research design assessed in the project**

We have pre-defined six categories that should encompass most clinical research.

- (Randomised) Controlled Trials (RCT) – where the aim is to assesses the effect of an intervention made by the research team against a comparator. Where a specific trial design is used, such as cluster methods, then this will be noted. We will include controlled trials with no clear randomisation. Pseudo-RCTs, where there has been no intervention by the research team, but rather existing data are used to make inferences about an intervention and comparator will be treated as observational.
- Diagnostic test accuracy (DTA) – where the aim is to assess the properties of a test, or measurement or clinical classification scheme.
- Observational studies (Observational) – further subdivided into case-study/case-series (authors own description), case-control, cohort and cross sectional, where the aim is to use existing data to look for patterns or associations, with no intervention by the research team. Within this rubric we will include case studies and case series.
- Qualitative (Qualitative) - where the aim is to interpret unstructured, non-numerical data to create meaning.
- Prognosis (Prognosis) – further subdivided into fundamental prognosis, prognostic factor, prognostic model or rule, where the aim is to assess for association between baseline states and future outcomes.
- Systematic review (SR) – where the aim is to use existing data from all relevant sources, usually aggregate published data in biomedical journals, to answer a research question. Other forms of meta-research will be included in this rubric.

Papers that do not fit any of these categories will be given a label of ‘other’.

**Additional File 1. Supplementary text S3.**

**Data dictionary for ‘included studies’ master sheet**

**Study ID:** unique identifier for each paper returned from search

**Journal:** Journal name. Data collection begins in the first month that the journal published eligible COVID-19 research

**Month:** Each journal has weekly print publications, so month and week are recorded using format ‘Month week x/4 [assuming a four-week month]’

**Author:** surname of first author

**Design:** The primary method used in the paper, described using the pre-defined categories of: Controlled trial, Observational, Test accuracy, Systematic review, Qualitative, Prognosis. If the paper does not fit any of these it will not be included.

**Case series:** Where the paper is described by the authors as a case-study or case series.

**Brief report:** Where the journal describes the submission as a brief report or similar, these would have less content than a full original research paper. Some journals have a regular section for brief reports and some publish such reports on an ad-hoc basis.

**Correction / retraction:** Where a substantial change to content is made following publication. This will be checked last week of May. Journals use differing approaches to highlighting corrections, but for all of our included journals, major corrections and retractions are linked to the online article. The Lancet has a ‘Department of Error’ section, where any corrections, including minor changes, are described. For the Lancet, we defined a major correction as more than one entry in the Department of Error section.

**Editorial:** Whether the paper was accompanied by an editorial in the same journal. Some of our included journals routinely publish comment on their included research, to quality as an editorial the comment must be labelled as such in the journal.

**COVID:** If the primary focus of the paper was COVID-19 / SARS COV-2 or the outcomes relate to COVID-19 / SARS COV-2.

**Topic:** The clinical discipline to which the paper belongs, using where possible MeSH subject headings.

**Exposure:** The intervention (controlled trial), the exposure (observational), the index test (test accuracy).

**Outcome:** The primary outcome (if no primary outcome is described, then coded as ‘various’)

**Total ‘N’:** The total population included at baseline or first assessment. For systematic reviews this is the number of included papers.

**Follow-up:** Time from first measure to last measure for the primary outcome (in weeks); for some study designs for example cross-sectional, systematic reiew this is coded as N/A

**Results:** In the abstract or main text conclusions, do the authors claim a 'significant result' that rejects the null, if so then label as 'positive'; if no then 'neutral'.

**Funding:** Who supported the study. Coded as academic or industry, if funding was mixed this is coded as industry.

**Additional File 1. Fig. S1-S4**

**Traffic light visual summary of risk of bias**

*In these visual summaries, green indicates low risk of bias, amber/yellow indicates uncertain risk of bias and red indicates high risk of bias. For some tools a blue category denotes ‘no available information and unable to score’.*

*Risk of bias is presented for differing study methods at individual study level and in aggregate*

**S1a) Traffic light summary of risk of bias for RCTs at individual study level**


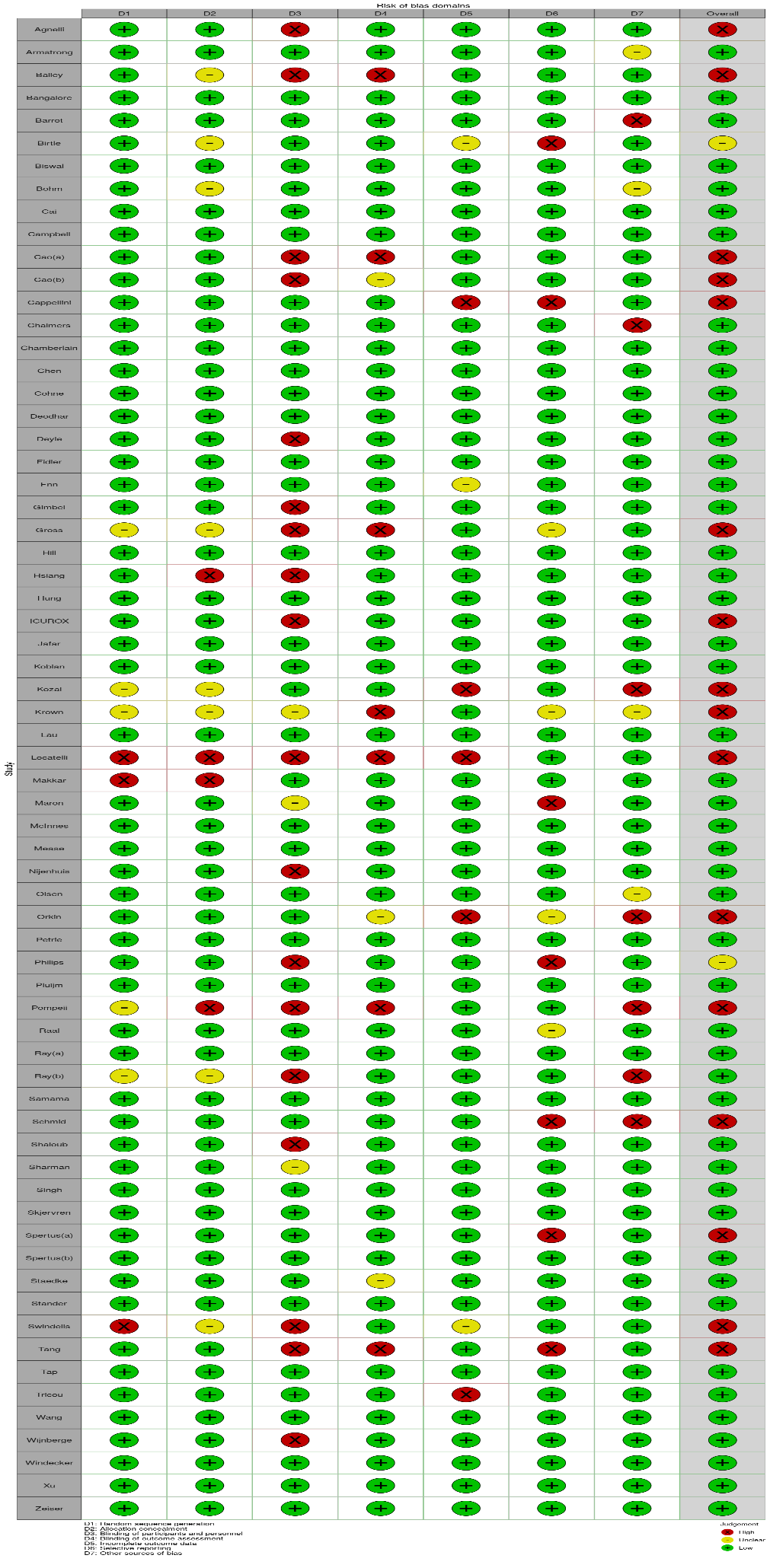


**S1b)** **Traffic light summary of risk of bias for RCTs as aggregate scores**


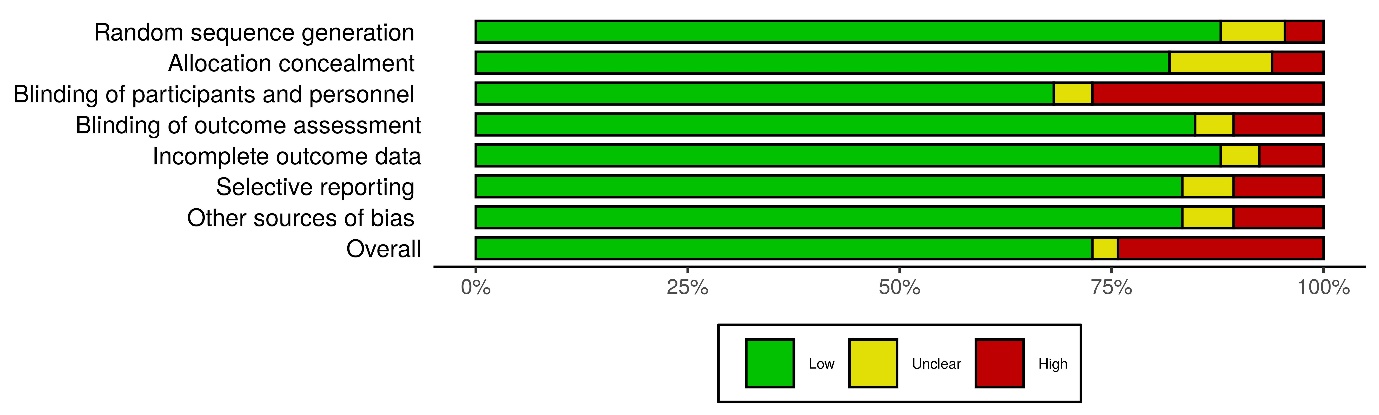


**S2a) Traffic light summary of risk of bias for observational studies at individual study level**


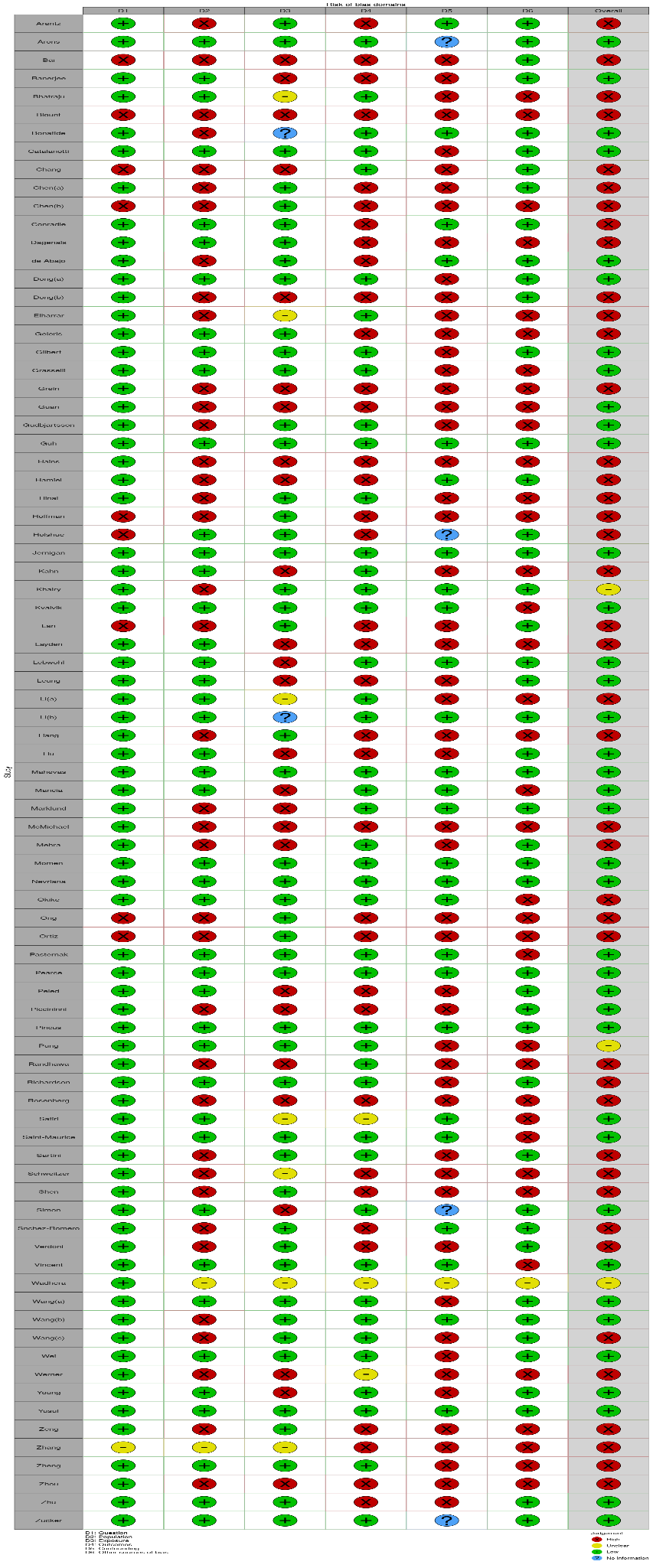


**S2b) Traffic light summary of risk of bias for observational studies at aggregate level**


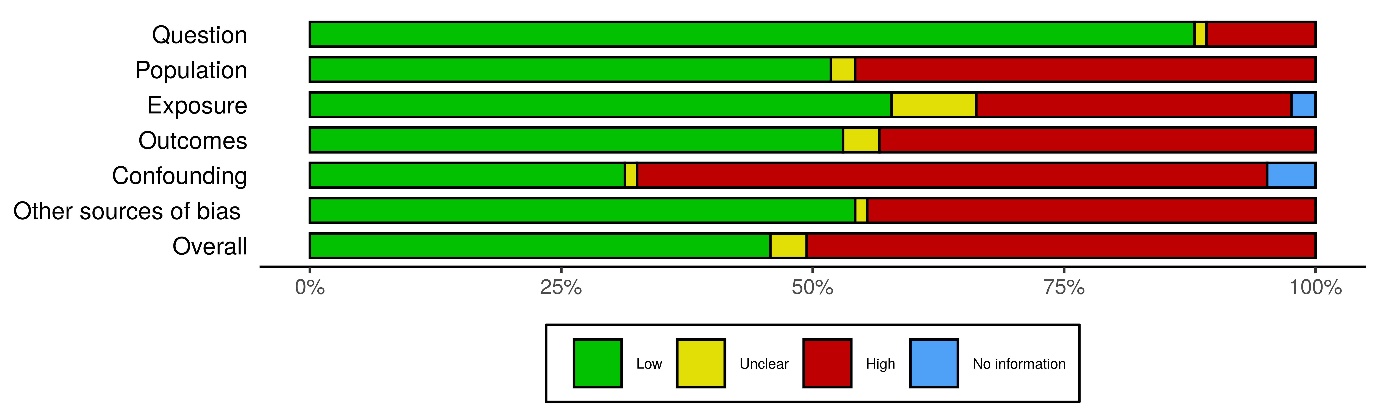


**S3a) Traffic light summary of risk of bias for test accuracy studies at individual study level**


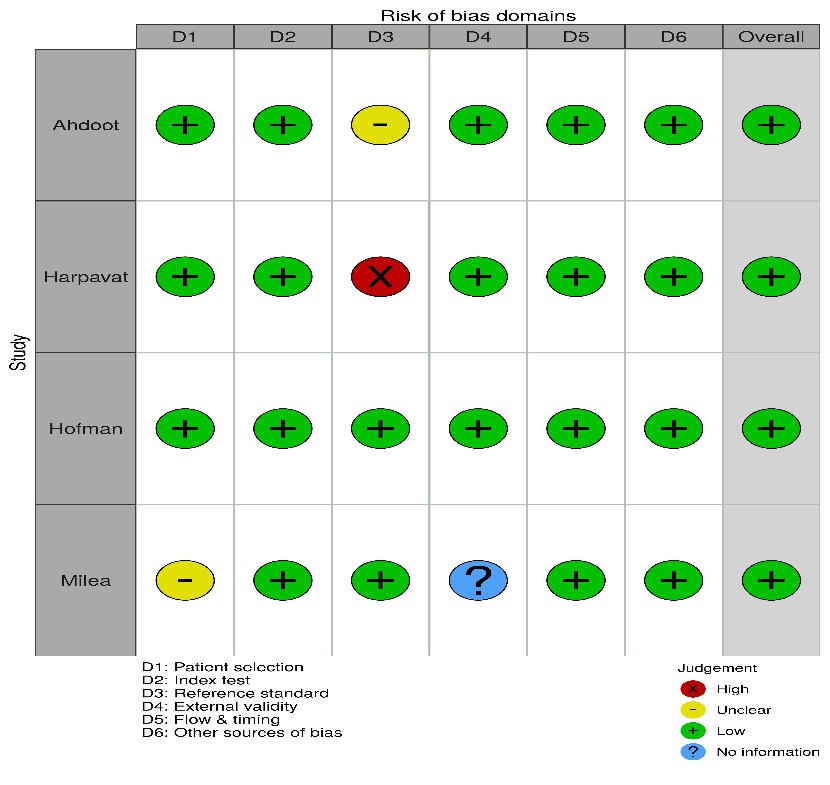


**S3b) Traffic light summary of risk of bias for test accuracy studies at aggregate level**


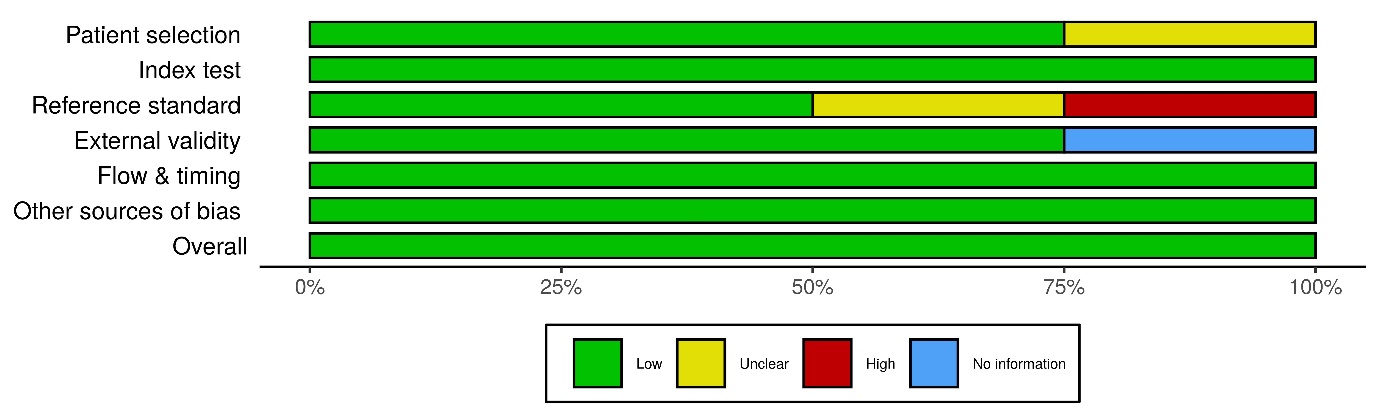


**S4a) Traffic light summary of risk of bias for systematic reviews at individual study level**


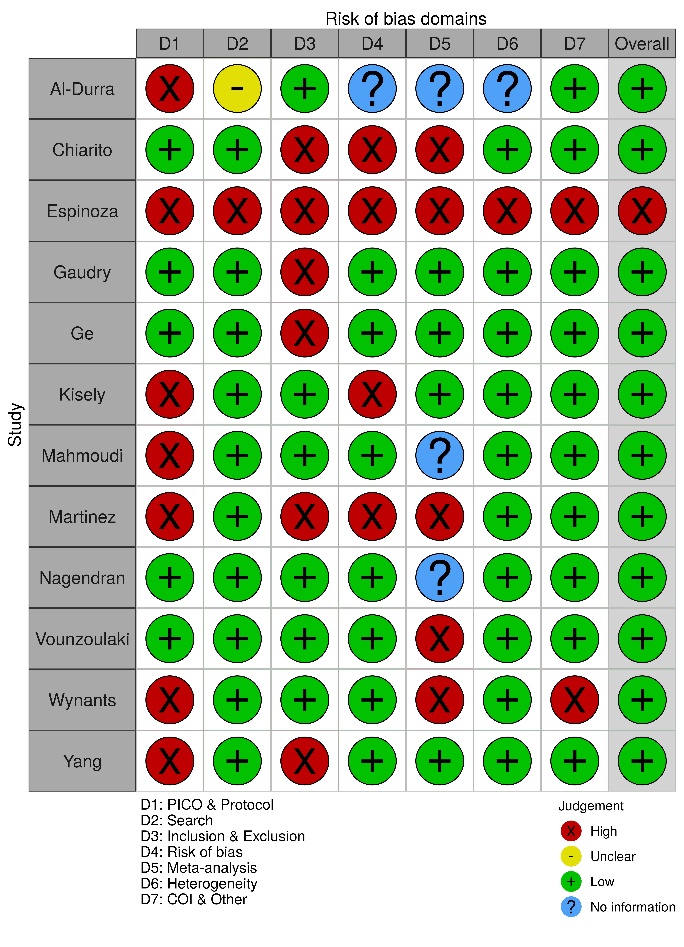


**S4a) Traffic light summary of risk of bias for systematic reviews at aggregate level**


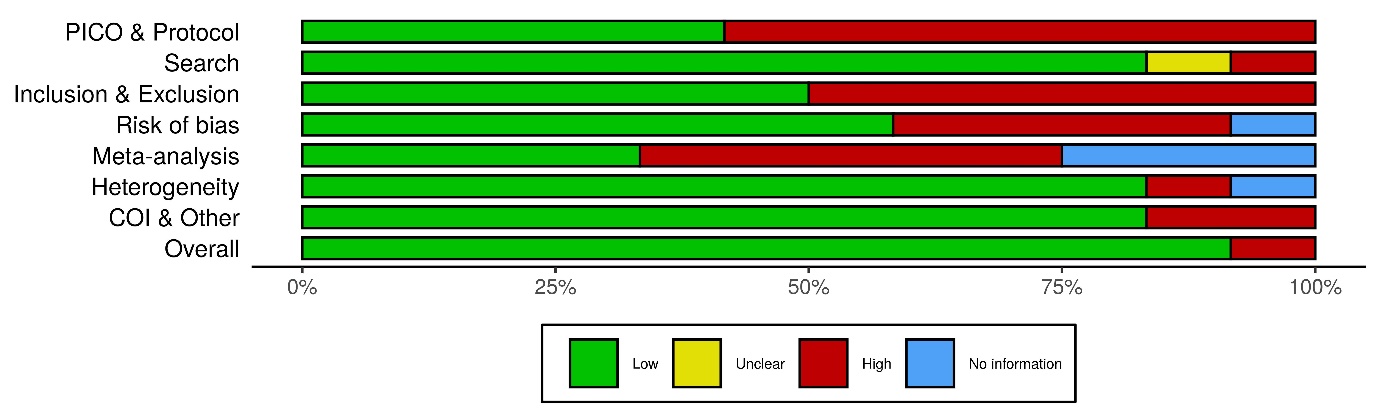


**Additional File 1. Fig. S5.**

**S5 Reporting guideline adherence (CONSORT) for randomised controlled trials**

*Green boxes indicate that the reporting suggestion was adhered to for that domain; amber boxes indicate that the reporting suggestion was not applicable for that particular study; red boxes indicate that the reporting suggestion was not adhered to.*

**Additional File 1. Fig. S6.**

**S6 Reporting guideline adherence (STROBE) for observational studies**

**Additional File 1. Fig. S7**

**S7 Reporting guideline adherence (STARD) for test accuracy studies**

**Additional File 1. Fig. S8**

**S8 Reporting guideline adherence (PRISMA) for systematic review**

**Additional File 1. Fig. S9**

**S9 Modified star plot describing overall and individual item level reporting adherence for STROBE (observational) reporting**

**
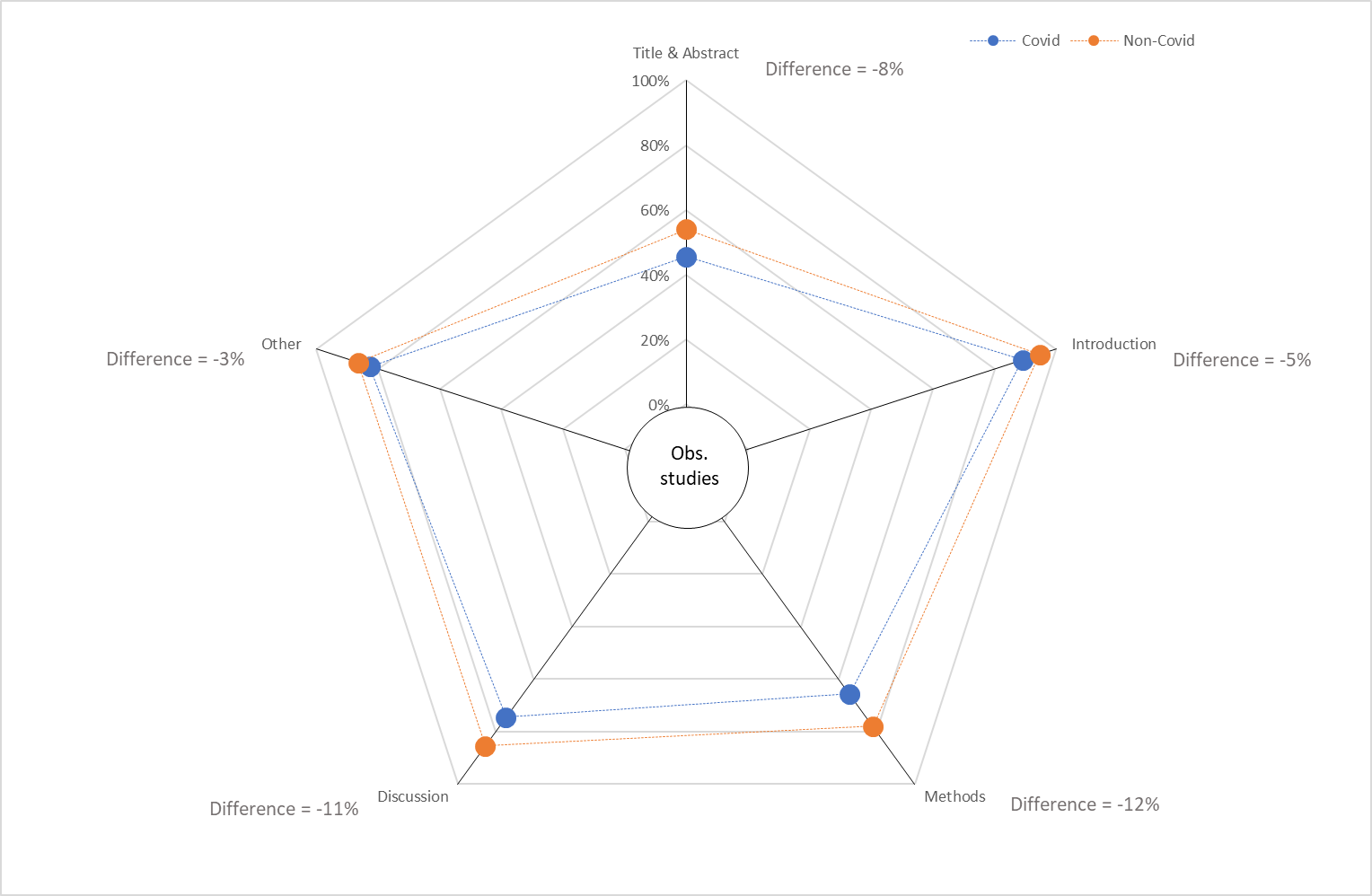
**

*Blue spokes represent covid-19 studies and orange spokes represent non-covid 19 studies.*

*‘RCTS’ = Randomised controlled trials ‘Obs studies = observational studies’*

**Additional File 1. Fig. S10**

**S10 Modified star plot describing overall and individual item level reporting adherence for CONSORT (RCT) reporting**

**
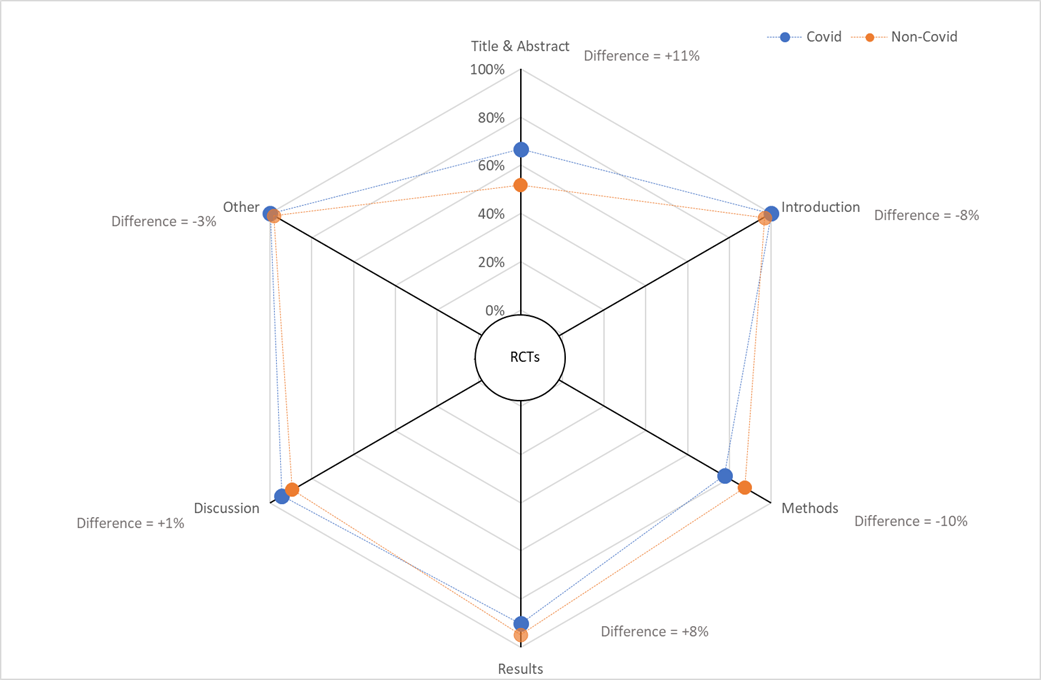
**

*Blue spokes represent covid-19 studies and orange spokes represent non-covid 19 studies.*

*‘RCTS’ = Randomised controlled trials ‘Obs studies = observational studies’*
